# Social and psychological factors affecting eating habits among university students in a Malaysian medical school: a cross-sectional study

**DOI:** 10.1186/1475-2891-11-48

**Published:** 2012-07-18

**Authors:** Kurubaran Ganasegeran, Sami AR Al-Dubai, Ahmad M Qureshi, Al-abed AA Al-abed, Rizal AM, Syed M Aljunid

**Affiliations:** 1 Department of Community Medicine, International Medical School, Management and Science University (MSU), Off Persiaran Olahraga, Section 13, 40100, Shah Alam, Selangor, Malaysia; 2 Community Medicine and Public Health, Cyberjaya University College of Medical Sciences, No. 3410, Jalan Teknokrat 3, Cyber 4, 63000, Cyberjaya, Selangor, Malaysia; 3 Community Health Department, Faculty of Medicine, Universiti Kebangsaan Malaysia (UKM), Jalan Yaacob Latiff, 56000, Cheras, Kuala Lumpur, Malaysia; 4 United Nations University- International Institute for Global Health, Jalan Yaacob Latiff, 56000, Kuala Lumpur, Malaysia

**Keywords:** Eating habits, Lifestyle, Malaysia medical students, Social and psychological

## Abstract

**Background:**

Eating habits have been a major concern among university students as a determinant of health status. The aim of this study was to assess the pattern of eating habits and its associated social and psychological factors among medical students.

**Methods:**

A cross sectional study was conducted among 132 medical students of pre-clinical phase at a Malaysian university. A self-administered questionnaire was used which included questions on socio-demography, anthropometry, eating habits and psychosocial factors.

**Results:**

Mean (±SD) age of the respondents was 22.7 (±2.4) years and (the age) ranged from 18 to 30 years. More than half had regular meals and breakfast (57.6% &, 56.1% respectively). Majority (73.5%) consumed fruits less than three times per week, 51.5% had fried food twice or more a week and 59.8% drank water less than 2 liters daily. Eating habits score was significantly low among younger students (18–22 years), smokers, alcohol drinkers and those who did not exercise. (*p*<0.05). Four psychological factors out of six, were significantly associated with eating habits (*p*<0.05). In multivariate analysis, age and ‘eating because of feeling happy’ were significantly associated with eating habits score (*p*<0.05).

**Conclusion:**

Most of the students in this study had healthy eating habits. Social and psychological factors were important determinants of eating habits among medical students.

## Background

Poor eating habits is a major public health concern among young adults who experienced transition into university life [1], during which, they are exposed to stress and lack of time [2,3]. These factors pose a barrier against adoption of healthy behaviors, such as poor eating habits and substance abuse [1]. Although these behaviors of students are considered temporary, as part of university life; unhealthy habits picked up at this age generally persist in older adult life [4].

Rapid changes in physical growth and psychosocial development have placed these young adults as nutritionally vulnerable groups with poor eating habits, that fails to meet dietary requirements [5-7]. Some common unhealthy eating patterns among young adults included meal skipping, eating away from home, snacking and fast food consumption [6,7].

Environmental factors also contribute to adoption of unhealthy eating habits among university students [8]. The mushrooming of shopping malls, convenience stores, vending machines and fast food outlets have created an alarming situation for young adults to practice unhealthy eating habits [9].

University students tend to make their own food choices [10] based on cost of food and availability of fast food [11]. They lack knowledge of healthy food choices that may affect eating habits and nutritional status negatively [11]. Previous studies revealed that university students failed to meet the recommended intakes of fruits and vegetables [12,13]. University students had frequent snacking habits [14] and had a higher frequency of fast food consumption [15].

It has been assumed that medical students would practice healthy dietary habits compared to non-medical students [16]. Some studies have found otherwise. A previous study in China revealed that medical students exhibited early risk factors for chronic diseases due to poor eating habits [17]. It was found that although medical students had sufficient knowledge regarding good dietary habits, they failed to apply this knowledge into practice [2]. Stress of university life and medical study load would be factors that negatively influence their diet [18].

In 2011, Gan et al. highlighted the presence of unhealthy eating behaviours and inadequate nutrient intake among university students [11]. The study concluded that there was a need to promote healthy eating habits among young adults to achieve a healthy nutritional status. Chin & Nasir (2009) [5] revealed that meal skipping; particularly breakfast, snacking and various weight loss dietary behaviours were some of the unhealthy eating behaviours depicted by Malaysian adolescent girls. The study concluded that promotion of healthy eating was crucial for future health well-being. There was no study in Malaysia that investigated the relationship between eating habits and the psychological factors among university medical students. The current study is aimed at assessing the patterns of eating habit and its associated factors, with focus on psychological factors among medical students in a Malaysian university.

## Methodology

### Study setting and population

This cross-sectional study was conducted among 140 medical students at a private university in Malaysia by using universal sampling. After arrangement with course co-coordinator and lecturers, students from the first year medical faculty were approached in the classroom after lectures. They were asked to participate in this study voluntarily. Objectives and benefits of the study were explained to respondents orally and in a written form attached to the questionnaire. They were assured that information obtained would be confidential and their participation would not affect their course progress. A written consent was obtained from those who agreed to participate. Approval of the study was obtained from the ethics committee of the University (approval number: JMS5/0182).

### Study instruments

We used a self-administered questionnaire on eating habits which was adopted from previous published studies [14,15]. The questionnaire consisted of three parts. The first part included questions on demographic data; such as age, gender, education level, marital status, ethnicity and living circumstances. Body mass index (BMI) and lifestyle; such as smoking, alcohol intake and exercise were also included in this part. The second part includes questions on eating habits and type of meals consumed (10 items), such as frequency of meals, type of meal, vegetables and fruits consumption, daily water intake, consumption of fast food, etc. The third part included questions on psychological factors that influenced dietary habits of respondents. Questions were selected from the validated Compulsive Eating Scale (CES) [16] that was used to measure uncontrolled eating patterns among college students; items included in this study were: “eat because of feeling lonely”, “feel out of control when eating”, “eat so much until stomach hurts”, “eat because of feeling upset or nervous”, “eat because of feeling bored” and “eat because of feeling happy”. The response options were ‘Yes’ or ‘No’.

### Statistical analysis

The Statistical Package for Social Sciences (SPSS) version 16.0 was used to analyse the data in this study. The BMI was calculated as weight in kilograms divided by height in square metres (kg/m^2^). In this study, based on the WHO BMI cut-offs for the Asian population, a BMI < 18.5 kg/m^2^ was categorised as underweight, 18·5–22·9 kg/m^2^ as the normal range, 23.0–27.4 kg/m^2^ as pre-obese, 27.5–34.9 kg/m^2^ as obese Class I, 35.0–39.9 kg/m^2^ as obese Class II and ≥ 40 kg/m^2^ as obese Class III [19]. To check for the validity of the Compulsive Eating Scale (CES) among the Malaysian population, an exploratory factor analysis was performed using principal component method with varimax rotation and Cronbach’s alpha was used to test the internal consistency of the scale. Each item of eating habits was scored (1) if the response was healthy or (0) if non healthy. All items were summed and the total score was obtained (minimum = 0 and maximum = 10). Thus, a higher score on eating habits indicated better eating habits. Descriptive analysis was performed for all variables. Student *t*-test and ANOVA test were used to compare mean eating habits across socio-demographic variables. Test of normal distribution of the total score of eating habits was also conducted. Hierarchical multivariate linear regression was used to obtain factors associated significantly with eating habit score. Age, working status of mother, drinking alcohol, exercise and smoking status were entered in the first step. In the second step, five out of six psychological factors affecting eating behavior were entered. Multicollinearity was checked between independent variables.

## Results

### Socio-demographic characteristics

One hundred and thirty two out of 140 students participated in this study with a response rate of 94.0%. The majority was females (70.5%) and aged more than 22 years (old 61.4%). Most of them were Malays (61.4%) while Indians and Chinese constituted (of) 31.8% and 2.3% respectively. Regarding mother’s education level, 44.7% had tertiary education, 37.9% had high school or less and the rest had non-formal education (17.4%). Regarding father’s education, majority had tertiary education (51.5%), 33.3% had high school or less and 15.2% had non-formal education. The majority of mothers were not working (57.6%). The majority had an average monthly household income of RM 3000 or less (59.1%) and living with their families (64.4%). The majority had denied smoking (94.7%) and alcohol consumption (97%). A lot of them performed regular exercise (78%), but some did not (22%). More than half (53%) had a normal BMI, 22.7% were under weights, 16.7% were pre-obese and 7.6% were obese Class I (Table 1).

**Table 1:** Socio -demographic characteristics of respondents (n = 132)

**Characteristics**	**N**	**%**
**Gender**		
Male	39	29.5
Female	93	70.5
**Age**		
18-21	51	38.6
≥ 22	81	61.4
**Ethnicity**		
Malay	81	61.4
Chinese	3	2.3
Indian	42	31.8
Others	6	4.5
**Mother’s education level**		
Non-formal education	23	17.4
High school or less	50	37.9
Tertiary education	59	44.7
**Father’s education level**		
Non-formal education	20	15.2
High school or less	44	33.3
Tertiary education	68	51.5
**Monthly household income (RM)**		
≤3000	78	59.1
3001-4999	22	16.7
≥5000	32	24.2
**Living arrangement**		
Living alone	47	35.6
Living with family	85	64.4
Body Mass Index (BMI)*		
Underweight (< 18.5)	30	22.7
Normal (18.5 – 22.9)	70	53.0
Pre-obese (23.0-27.4)	22	16.7
Obese class I (27.5-34.9)	10	7.6
**Mother working**		
Yes	56	42.4
**Smoking**		
Yes	7	5.3
**Alcohol**		
Yes	4	3.0
**Regular exercise**		
Yes	103	78

* BMI is calculated based on WHO criteria for Asian population.

### Eating habits

More than half took meals and breakfast regularly (57.6%, 56.1% respectively). About 57.6% had snacks less than three times per week and 42.4% took snacks three or more times per week. The majority consumed vegetables and legumes three or more times per week (81.8%). Almost half of them (51.5%) consumed fruits less than three times per week; the rest (48.5%) took it three times or more. Many had fried food twice a week or more (73.5%), while 26.2% took it less than two times. The majority (78.8%) had fast food rarely and took meals with family or friends daily (81.1%). Most of them had a balanced variety of foods (60.6%) while 18.9% preferred meat and 5.3% preferred vegetables. The majority had less than two liters water intake daily (59.8%) (Table 2).

**Table 2:** Eating habits among respondents (n = 132)

**Characteristics**	**N**	**%**
**Regular meals**		
Yes	76	57.6
No	56	42.4
**Daily breakfast**		
Yes	58	43.9
No	74	56.1
**Frequency of daily meals**		
Less than three times	79	59.8
Three or more times	53	40.2
**Frequency of having snacks** (per week)		
Less than three times	76	57.6
Three or more times	56	42.4
**Weekly consumption of vegetables & legumes**		
Less than three times	24	18.2
Three or more times	108	81.8
**Weekly consumption of fruits**		
Less than three times	68	51.5
Three or more times	64	48.5
**Weekly consumption of fried food**		
Less than twice	35	26.5
Twice or more	97	73.5
**Consumption of fast food**		
Often	28	21.2
Rarely	104	78.8
**Meals with friends & family**		
Daily	107	81.1
Not daily	25	18.9
**Type of food consumed**		
Mainly meat	25	18.9
Mainly vegetables	7	5.3
Carbohydrate (rice, bread)	20	15.2
Variety of food in balance	80	60.6
**Water intake (liters/day)**		
< 2	79	59.8
≥2	53	40.2

### Psychological factors affecting eating behavior

Cronbach’s alpha coefficient of the Compulsive Eating Scale (CES) was 0.80. The exploratory factor analyses yielded one factors with given values greater than 1 (3.1). The two-factor solution accounted for 51.0% of the variance. Factor loading ranged from 0.41 to 0.50.

Nearly 48.5% ate because of feeling lonely, 62.1% felt completely out of control when it comes to food, 53.8% ate till stomach hurts, 53% ate because of feeling upset or nervous and 59.1% ate because of feeling bored. The majority ate because of feeling happy (80.3%) (Table 3).

**Table 3:** Psychological factors affecting respondents eating habits among respondents (n = 132)

**Psychological factors**	**Yes n (%)**	**No n (%)**
Eat because of feeling lonely	64 (48.5)	68 (51.5)
Feel completely out of control when it comes to food	82 (62.1)	50 (37.9)
Eat so much until stomach hurts	71 (53.8)	61 (46.2)
Eat because of feeling upset or nervous	70 (53.0)	62 (47.0)
Eat because of feeling bored	78 (59.1)	54 (40.9)
Eat because of feeling happy	106 (80.3)	26 (19.7)

### Association between eating habits and socio-demographic factors

Mean total score of eating habit for all the participant was 6.3(SD ± 1.8) and ranged from 2 to 10. Mean with (SD) total score of eating habits was compared across the categorical variables in the study. Mean for those aged ≥22 years and those aged 18–21 years was 6.68(SD ±1.66) and 5.86 (SD ± 1.87) respectively and this difference was significant (*p* = 0.01). Significant difference in eating habits score was also found between smokers, 4.86 (SD ± 1.57) and non smokers, 6.45 (SD ±1.76), (*p* = 0.02) and between those who drank alcohol, 4.25 (SD ±2.06) and those who did not, 6.43(SD ±1.74), (*p* = 0.02). No significant association was found between eating habits and other socio-demographic factors (Table 4).

**Table 4:** Association between eating habits score and categorical variables (n = 132)

**Categorical variable**		**Mean(SD)**	***p*value**
**Gender**	Male	6.28 (1.82)	
	Female	6.40 (1.77)	0.73
**Age**	18-21	5.86 (1.87)	
	≥ 22	6.68 (1.66)	0.01
Ethnicity*	Malay	6.31 (1.81)	
	Chinese	8.33 (1.53)	
	Indian	6.33 (1.75)	
	Others	6.33 (1.51)	0.29
Mother’s education level *	Non-formal education	6.99 (1.56)	
	High school or less	6.32 (1.58)	
	Tertiary education	6.19 (1.99)	0.25
Father’s education level *	Non-formal education	7.05 (1.76)	
	High school or less	6.32 (1.68)	
	Tertiary education	6.19 (1.99)	0.16
Monthly household income*	≤3000	6.36 (1.71)	
	3001-4999	6.23 (1.54)	
	≥5000	6.47 (2.11)	0.88
**Living arrangement**	Alone	6.40 (1.79)	
	With family	6.30 (1.78)	0.75
**Mother working**	Yes	6.04 (1.61)	
	No	6.61 (1.87)	0.07
**Smoking**	Yes	4.86 (1.57)	
	No	6.45 (1.76)	0.02
**Alcohol**	Yes	4.25 (2.06)	
	No	6.43 (1.74)	0.02
**Regular exercise**	Yes	6.51 (1.81)	
	No	5.86 (1.60)	0.09
Body Mass Index (BMI)*	Underweight (< 18.5)	6.23 (1.57)	
	Normal (18.5 – 22.9)	6.47 (1.90)	
	Pre-obese (23.0-27.4)	6.68 (1.67)	
	Obese class I (27.5-34.9)	5.30 (1.49)	0.20

* One way ANOVA test was used to compare mean between categories.

### Association between eating habits and Psychological factors

Mean with (SD) of total score of eating habit was compared between those who answered ‘yes’ and those who answered ‘no’ on each item of the psychological factors. Mean total score of eating habit for those who ate when lonely was 5.95 (SD ±1.78) and for those who did not was 6.75 (SD ±1.70) (*p* = 0.01). Mean for those ate till stomach hurt was 6.06 (SD ±1.76), and for those who did not was 6.72 (SD ±1.74) (*p* = 0.03). Mean for those who ate when upset and those who did not was 6.07 (SD ± 1.75) and 6.69 (SD ± 1.77) respectively (*p* = 0.04). Mean for those who ate when bored was 5.91 (SD ±1.67) and for those who did not was 7.02 (SD ± 1.74) (*p*<0.01) (Table 5).

**Table 5:** Association between eating habits score and psychological factors (n = 132)

**Psychological factors**	**Mean(SD)**		***p*value**
	**Yes**	**No**	
Eat because of feeling lonely	5.95 (1.78)	6.75 (1.70)	0.01
Feel completely out of control when it comes to food	6.32 (1.85)	6.44 (1.08)	0.70
Eat so much until stomach hurts	6.06 (1.76)	6.72 (1.74)	0.03
Eat because of feeling upset or nervous	6.07 (1.75)	6.69 (1.77)	0.04
Eat because of feeling bored	5.91 (1.67)	7.02 (1.74)	<0.01
Eat because of feeling happy	6.31 (1.82)	6.58 (1.63)	0.50

### Factors associated with eating habits in the hierarchical multiple linear regression

Age, working status of mother, drinking alcohol, exercise and smoking status were entered in the first step. In the second step, the following factors were entered: “eat because of feeling lonely”, “feel out of control when eating”, “eat so much until stomach hurts”, “eat because of feeling upset or nervous” and “eat because of feeling happy”. The results from the first step indicated that age was significantly associated with eating habits score (*p* = 0.006). In the second step, factors associated with eating habits score were age (*p* = 0.009), drinking alcohol (p = 0.037) and eat because of feeling happy *(p* = 0.009) (Table 6). The total model was significant (*p*<0.001) and accounted for 19% of the variance. There was no multicollinearity between variables.

**Table 6:** Results of the hierarchical multiple linear regression; factors associated with eating habits score (n = 132)

	***Step 1***			***Step 2***		
	**B**	**Beta**	***p*value**	**B**	**Beta**	***p*value**
≥ 22 years old	0.675	0.238	0.006	0.629	0.222	0.009
Mother working	0.344	0.096	0.264	0.283	0.079	0.349
Drinking alcohol	1.514	0.146	0.139	2.090	0.202	0.037
Exercise	0.619	0.145	0.083	0.546	0.128	0.121
Smoking	0.906	0.115	0.251	0.394	0.050	0.613
Eat because of feeling lonely				0.229	0.065	0.478
Eat because of feeling out of control when eating				0.544	0.149	0.096
Eat so much until stomach hurts				0.410	0.115	0.199
Eat because of feeling upset or nervous				0.093	0.026	0.778
Eat because of feeling happy				0.931	.258	0.009

The reference group for age is ‘18-21 years’; for exercise is ‘no’; for all other variables is ‘yes’.

## Discussion

In this study, more than half of respondents had meals regularly and 40.2% had meals of at least three times per day. This finding was comparatively lower than that reported by a Chinese study in which 83.6% of university students consumed regular meals, with 79% of them took at least three times per day [17]. Another study reported that 61.4% of Lebanese university students had regular meals daily[14].

Regular breakfast consumption among medical students is important for sufficient energy intake to overcome fatigue due to busy (daily) learning schedule [20]. In this study, less than half of respondents (43.9%) had breakfast daily. This finding was higher in comparison to a previous study [14] which found that 31.8% of study population had breakfast daily. However, some studies from Malaysia found higher rates of daily breakfast consumption among Malay undergraduate students in Selangor (75.6%) [21] and female adolescents in Pahang (52.6%) [5].

The frequent consumption of snacks and light meals is a recognizable aspect of teenage food behavior [22]. Surprisingly, our study found that only 42.4% of respondents had snacking at least three times per week. This finding was comparatively lower than previous studies from different countries, which found greater proportion of Syrian adolescents (53.0%) and Lebanon students (53.2%) [14,23] consumed snacks regularly.

The majority of respondents in our study consumed vegetables and legumes frequently (81.8%). This finding was high in comparison to previous studies from China (47.9%) [17] and Bahrain (26.3%) [22]. However, one study from Malaysia found that only 19% of university students consumed vegetables more than three times per week [11]. Our study also found that 48.5% of respondents consumed fruits at least three times per week. Similar finding was reported by Yahia et al.,(2008) [14]. It was reported that low intake of fruits and vegetables is associated with several chronic diseases at adulthood [24]. Our study disclosed that majority of medical students were aware of this health risk.

The typical university student diet is usually high in fat [25]. Students often select fast food due to its palatability, availability and convenience [14]. Surprisingly, our study found that only 21.2% of respondents consumed fast food often. Chin and Nasir, (2009) [5] reported that only 4.7% of respondents visited fast food restaurants frequently. In contrast, Moy et al., (2009) [12] reported that 60-70% of primary school students were fond of fast food. However, our study also found that majority of respondents (73.5%) consumed fried food at least twice a week or more, which was in line with that found by a previous study [14].

Most of the respondents in this study (81.8%) had meals with their family or friends. This is comparatively higher to that found by a previous study in which 42.7% of university students had meals with their families or peers [14].

Smoking and alcohol consumption were significantly associated with eating habit in this study. Similar findings were reported among Chinese university students [17]. Our study also found a significant association between age and eating habits.

Attending a university or college can be a stressful experience for many college students [26]. Previous studies found that behavioral consequences of stress may affect eating habits [27,28]. People living in a stressful society tend to eat more as a way of coping with stress [26]. A possible new innovation in this study was the association between eating habits and psychosocial factors among Malaysian medical students; eating habits score in this study was significantly lower among those who answered ‘yes’ on the following statements: “eat because of feeling lonely”, “eat until stomach hurts”, “eat because of feeling upset or nervous” and “eat because of feeling bored”. Kagan & Squires, (1984) [16] suggested that uncontrolled eating patterns among college students could be due to compulsive eating behaviors. With the paradigm shift towards industrialization and cultural change globally, information on healthy diet has become scarce in many developing and developed nations. The most vulnerable group, being university students, have adopted unhealthy eating behaviors due to reduced availability, affordability and accessibility of healthy diet in university campuses and surrounding food outlets. This study exhibited multi-factorial causes affecting eating habits among Malaysian university students. Understanding the contexts of such multi-factorial causes may help healthy food promotional activities by parents, university authorities, food providers and health promotion officers. Results of this study may help to create a foundation for possible interventional programs on healthy eating habits promotions. Blended with different socio-cultural and psychological attributes across different regions, a unified healthy eating policy should be drafted, being potentially amalgamated and practiced in all regions including developing and developed nations.

## Conclusion

In general, most of the students in this study had healthy eating habits except in frequency of meals, fruit consumption, water intake and consumption of fried food.

Social and psychological factors were important determinants of eating habits among medical students. Nutritional education among medical students should be encouraged to promote healthier eating habits and lifestyles, as well as adherence to the healthier traditional food. It is recommended that the scope of future research should be broadened to include a larger representative sample size of medical students by including students from different medical colleages from all Malaysia.

## Abbreviations

CI, Confidence interval; OR, Odds ratio; CES, Compulsive Eating Scale; BMI, Body Mass Index; SD, Standard Deviation.

## Competing interests

The authors have no competing interests to declare.

## Authors’ contributions

SAR and KB designed the research study. AMQ and AAA conducted the data entry, data cleaning and descriptive analysis. SAR and KB were responsible for data analysis and interpretation of results. SAR, KB and AMQ wrote the paper. RAM and SMA revised the final draft critically for important intellectual content. All authors read and approved the final manuscript.
